# Medicated Cigarettes

**Published:** 1863-10

**Authors:** W. E. Bowman


### MEDICATED CIGARETTES.

By W. E. BOWMAN, M. D.

Cigarettes may be made of almost any variety of thick paper, but that kind should be selected that on burning yields a smoke most easily inhaled. I have always employed the heavy paper used for copy book covers (olive pressings); thick blotting paper, however, makes a good cigarette, but the regular filtering paper does not answer, as its smoke is dense and suffocating.

First, cut the paper into strips about seven inches long and an inch and a quarter wide, and next ascertain how much fluid it requires to saturate twenty-five of these pieces. This is readily done by soaking them in an exactly measured ounce of water, when on withdrawal it will be found that about five fluid drachms of the liquid has been imbibed; this will give the key to the strength you are to make the solutions.

Next saturate the slips with the remedy, and when nearly dry gum or paste one border of each, and roll it around a pencil spirally, afterwards withdraw the pencil, and the cigarette is made.—*Can. Lan.*

*Arsenical Cigarettes.*—Boil 25 grains of arsenious acid (the lump broken up is purest) in a Florence flask, with four ounces of water, down to the quantity required to saturate 100 slips of paper previous to rolling. They will then contain a quarter of a grain each. If you have not the usual apparatus, hang the flask above some live coals by means of a wire.

*Mercurial Cigarettes.*—Dissolve three drachms of red precipitate in three drachms of nitric acid, and add enough water to make up the quantity required to saturate 100 slips of paper. They will contain about three grains of the nitrate of mercury.

*Nitre Cigarettes.*—Dip the paper in a saturated solution of the nitrate of potash before rolling.

*Balsamic Cigarettes* are made by giving the dried nitre cigarettes a coating of tincture of benzoin.

In the *British Medical Journal*, Dr. Nevins, of the Royal Infirmary School of Medicine, Liverpool, speaks highly of these cigarettes in a number of cases.

*Aphonia.*—A patient who could not speak above a whisper for over a year, probably due to a thickened condition of the chordæ vocales, as she had no pain or constitutional symptoms, used the mercurial cigarettes for a month, and perfectly recovered.

*Offensive Discharges from the Nostrils*, with a sense of uneasiness in the frontal sinuse, was quite cured in about a month with the mercurial cigarettes. The patient held his nose after taking a mouthful of the smoke, and then forced it into his nostrils in the manner practiced by accomplished smokers.

*Polypus in the Nose.*—A patient who had been twice operated upon for polypus, is now able to keep the disposition to form fresh polypi in check, by smoking the mercurial cigarette in the same manner, when he feels that uneasiness which warns him of the danger of its recurrence.

*Deafness.*—When dependent upon an obstructed Eustachian tube, he finds the nitre cigarettes, made with brown paper, most successful, and that the smoke forced into the tympanum from the throat gradually restores the sense of hearing. The circumstance which first led him to adopt this method, was hearing a deaf person on one occasion remark, that when he was sneezing the day before, he heard perfectly; the violent effort appeared for the moment to have dilated the Eustachian tube, and hearing was the result. He says, that in a deafness of seven years' standing, he had benefitted a patient more by this treatment than by any other.

*Phthisis.*—Trousseau long ago recommended a puff or two of an arsenical cigarette twice or three times a day in phthisis.

When the attention of the profession has been duly aroused to this subject, there will doubtless be found many other affections in which medicated cigarettes may be advantageously employed, as in syphilitic ulcerations of the throat, ozæna, offensive breath, obstruction of the lachrymal duct, diphtheria, &c., &c.—*Canada Lancet.*

